# Urological manifestations in familial Mediterranean fever excluding renal amyloidosis: a systematic review

**DOI:** 10.1093/rheumatology/keag127

**Published:** 2026-03-24

**Authors:** Aziz Kutay Ozcan, Baris Karaoglan, Berkay Gundogdu, Cansu Sahin, Muhammed Fatih Simsekoglu, Serdal Ugurlu

**Affiliations:** Department of Internal Medicine, Cerrahpasa Medical Faculty, Istanbul University-Cerrahpasa, Istanbul, Turkey; Department of Internal Medicine, Cerrahpasa Medical Faculty, Istanbul University-Cerrahpasa, Istanbul, Turkey; Department of Internal Medicine, Cerrahpasa Medical Faculty, Istanbul University-Cerrahpasa, Istanbul, Turkey; Department of Internal Medicine, Cerrahpasa Medical Faculty, Istanbul University-Cerrahpasa, Istanbul, Turkey; Department of Urology, Cerrahpasa Medical Faculty, Istanbul University-Cerrahpasa, Istanbul, Turkey; Division of Rheumatology, Department of Internal Medicine, Cerrahpasa Medical Faculty, Istanbul University-Cerrahpasa, Istanbul, Turkey

**Keywords:** familial Mediterranean fever, urological manifestations, urologic diseases, testicular diseases, epididymo-orchitis, acute scrotum, testicular torsion, infertility, amyloidosis, genitourinary manifestations

## Abstract

**Objectives:**

To systematically review the spectrum of urological manifestations and fertility outcomes reported in FMF patients, excluding renal amyloidosis–related involvement.

**Methods:**

A systematic search was conducted according to PRISMA 2020 Guidelines in PubMed, Web of Science, Google Scholar and Cochrane Library, up to 4 July 2025. Studies that included FMF patients with a confirmed urological pathology were eligible. Data was extracted and presented through descriptive statistics.

**Results:**

A total of 110 records were published between 1973 and 2025, of which 38 met the inclusion criteria (14 case reports, 10 case series, 10 cross-sectional and 4 cohort studies) and covered a total of 2040 patients (mean ± s.d. age 23.35 ± 16 years; 43.2% female). Results included acute scrotum (*n* = 64) with 75% of recurrence, testicular amyloidosis (*n* = 40) confirmed with biopsy, epididymo-orchitis (*n* = 25) with fever present in 84%, testicular torsion (*n* = 4), hydrocele (*n* = 4), and bladder amyloidosis (*n* = 2). Among 189 semen analyses reported, azoospermia and oligospermia were in 26.9% and 14.8% of cases. Infertility was reported in patients with testicular amyloidosis.

**Conclusion:**

Our study results show that acute scrotum and epididymo-orchitis were the most frequent urological manifestations in FMF, and testicular amyloidosis and fertility impairment were also notable. These findings highlight the importance of considering urological involvement as part of the FMF spectrum in clinical practice.

## Graphical abstract

**Graphical Abstract:**
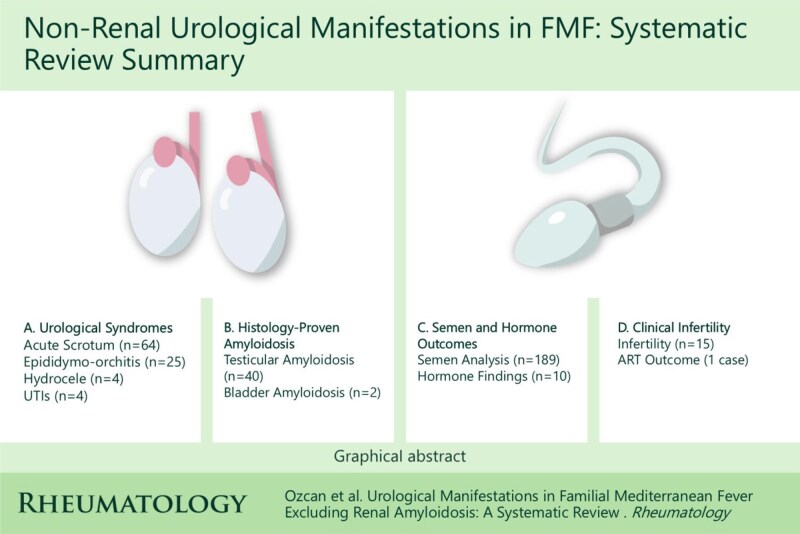
This graphical abstract summarizes the findings of our systematic review on non-renal urological manifestations in FMF. The figure visually categorizes these manifestations into four domains—urological syndromes, amyloidosis, semen/hormone findings and infertility—to provide a concise overview

Rheumatology key messages

- Urological manifestations of FMF, predominantly acute scrotum, are under-recognized but clinically relevant.
- Symptom driven scrotal US sonography and semen analysis should be considered if patient is affected.
- Children presenting with recurrent acute scrotum should be evaluated for FMF in the differential diagnosis.

## Introduction

FMF is an autoinflammatory disease, resulting from point mutations in the *MEFV* gene on chromosome 16 [1]. These mutations lead to increased pyrin activation and dysregulated IL-1β production, resulting in inflammation [2]. The characteristic features of FMF are serositis of the pleura and peritoneum, arthritis, synovitis and recurrent febrile episodes lasting up to 4 days. These symptoms typically respond to colchicine therapy [3].

Although musculoskeletal and serosal manifestations of FMF are well recognized [4], the disease may also affect the genitourinary system, a manifestation that is underreported yet clinically important. The tunica vaginalis testis, a serosal derivative of the peritoneum, covers the epididymis and testis in a double-layered serous membrane, and thus represents an anatomical site prone to inflammation [5]. Although several studies have described bladder, testicular and epididymal pathology, the overall evidence base remains limited [6–8].

Several mechanisms may underlie the urologic manifestations. Chronic inflammation and amyloid deposition may damage the testicular and urinary tract tissues, thereby contributing to impaired spermatogenesis and infertility [9]. Recurrent inflammatory episodes could affect the scrotal serosa leading to acute scrotum [6]. Moreover, systemic inflammation may disrupt hormonal balance, compounding these effects [10].

This review aims to compose and discuss published reports on urological manifestations, including fertility issues, as well as urogenital signs and symptoms associated with FMF, excluding renal amyloidosis–related involvement to focus on non-renal urological complications, and to highlight their clinical and diagnostic relevance with the aim of improving recognition and management.

## Methods

This systematic review was conducted according to the Preferred Reporting Items for Systematic Reviews and Meta-Analyses (PRISMA) 2020 guidelines [11].

### Registration and protocol

This review has been registered in PROSPERO (Registration ID: 1237303). The protocol was registered retrospectively; however, it corresponds to the original methodological approach. Any protocol deviations are reported in the manuscript.

### Eligibility criteria

Eligibility criteria for this systematic review included patients with FMF who had other urological signs or symptoms, including acute scrotum, epididymo-orchitis, testicular torsion and infertility. Due to the possibility of systemic diseases affecting the kidneys, amyloidosis outside the urinary tract (including the kidneys), as well as haematuria or proteinuria without urological manifestations, were excluded. To enable a more objective assessment of fertility and sperm parameters, the maximum age was set at 45 years. Other than fertility, there were no age constraints for patients.

All available English-language studies, without a start date restriction, up to 4 July 2025, were included. The first included study was published in 1973 [12], and the most recent was published in 2025 [13]. After excluding duplicates and irrelevant studies by title, 110 studies remained, including reviews, comparative studies, clinical trials, case reports, case series and others. Thirty-eight studies were included after full-text review and were categorized as case series, cohort, cross-sectional and case reports.

### Information sources

The search was performed in the Cochrane Library, Web of Science, Google Scholar and PubMed. Due to differences among the search engines, search terms were manually adapted as necessary (Supplementary Table S1).

### Search strategy

The MeSH terms were designed to identify studies related to FMF and urological diseases, testicular diseases, epididymitis, orchitis, male infertility and bladder diseases (including urinary tract, bladder, urological, epididymo-orchitis, acute scrotum, scrotal pain, testicular torsion, testicular infection, infertility, sterility and subfertility).

As Google Scholar has limited support for MeSH terms and Boolean operators, the search strategy was adapted accordingly.

Detailed search strategies for each database are provided (Supplementary Table S1).

### Selection process

**Figure 1:**
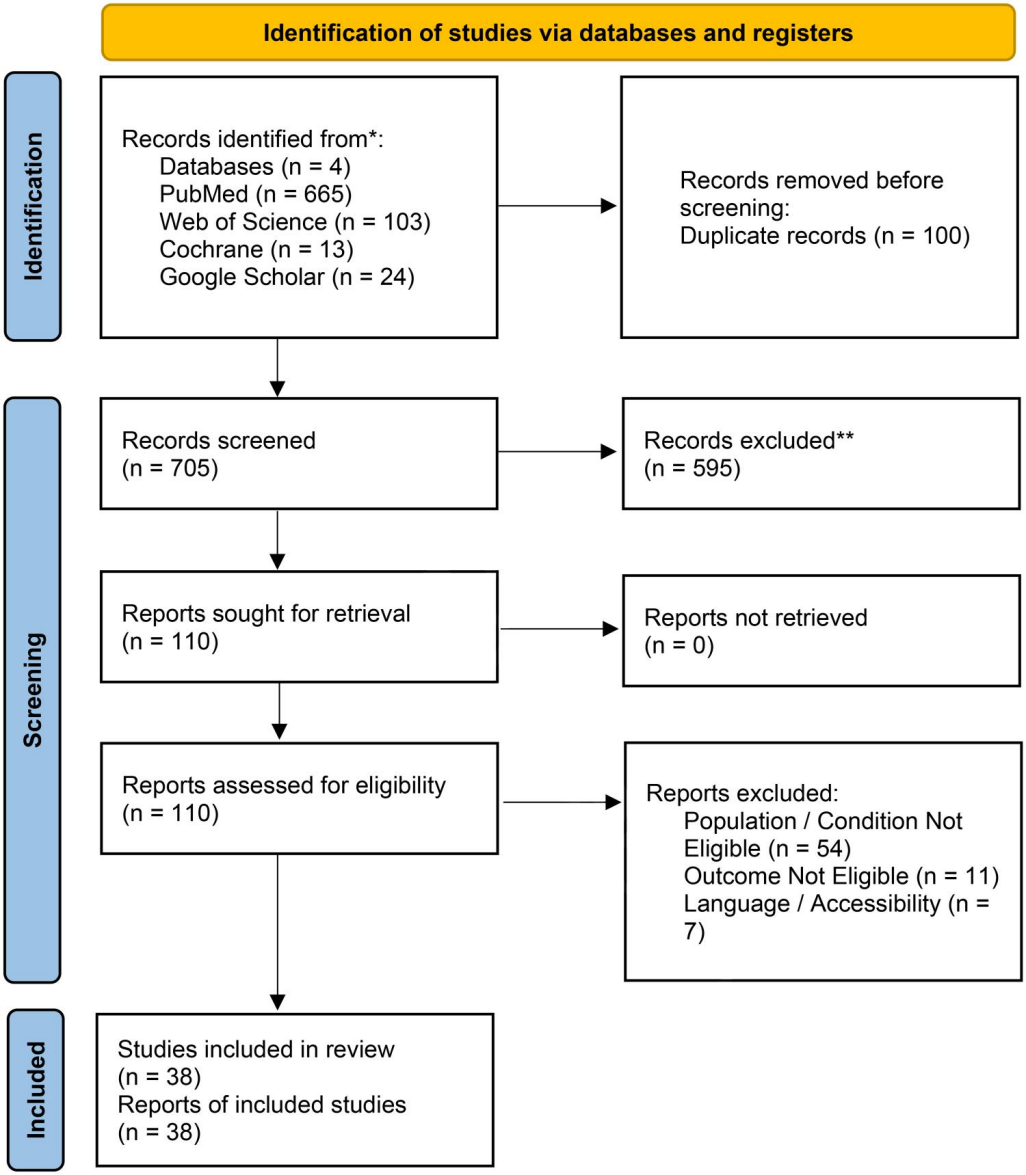
PRISMA 2020 flow diagram of the study selection process. This diagram illustrates the identification of records from four databases, removal of duplicates, screening of titles and abstracts, full-text eligibility assessment with reasons for exclusion, and inclusion of 38 studies in the final analysis. PRISMA: Preferred Reporting Items for Systematic Reviews and Meta-Analyses

Studies were excluded when they did not primarily involve FMF patients or presented a non-compatible diagnosis, focused on renal amyloidosis as the main outcome, lacked FMF-related urological manifestations, evaluated urological conditions unrelated to FMF, or were inaccessible due to language or inability to retrieve the full text (Fig. 1). Previous reviews were also removed to avoid duplicates, but their reference lists were screened to identify additional eligible studies. Four researchers independently searched all databases while two researchers conducted the Google Scholar search. All included studies were re-evaluated by two researchers to minimize bias.

### Data collection process

After selecting 38 studies to review for data extraction and statistics, they were divided among four researchers. Each researcher collected data independently on shared Google Sheets (Google LLC). The data were then cross-checked collaboratively, and statistical analyses were performed using IBM SPSS Statistics version 26.0 (IBM Corp., Armonk, NY, USA).

### Data items

Parameters were extracted from the studies, and categorized into 11 domains:

- Study identification: studies were first identified, and information on research centres and patients’ countries was recorded.
- Patient identification: race, age and gender were collected.
- FMF information: including FMF history, family history, amyloidosis and any related conditions.
- Medical treatment: laboratory findings, including markers of sterile inflammation and reported hormonal changes, were extracted.
- Laboratory results: blood and hormonal changes were present in the studies and were evaluated.
- Imaging and biopsy: imaging techniques [US sonography (USG), MRI, CT] and biopsy findings were reviewed to assess their role in detecting FMF-related urological conditions.
- Fertility: information on fertility, infertility and sterility was extracted.
- Semen analysis: semen analyses were collected when available
- Urological information: urological symptoms (scrotal pain, swelling, torsion, epididymitis, orchitis and urinary symptoms) were recorded. Acute scrotum was defined as an acute scrotal presentation (epididymitis, orchitis, epididymo-orchitis, testicular torsion or acute hydrocele), whereas specific diagnoses were preserved as reported by each study [14].

When age was described categorically, approximate values were assigned (infancy, 0.5; childhood, 7; ‘patients younger than 16’, 12), following the recommendation in the Cochrane Handbook Systematic Reviews of Interventions [15]. As only four cases were described qualitatively, this approach was preferred over maintaining qualitative categories.

### Study risk of bias assessment

All the studies were evaluated according to the Joanna Briggs Institute (JBI) Critical Appraisal Tool [7]. JBI checklists were used to assess reporting adequacy rather than overall risk of bias, given the nature of the included study designs.

### Effect measures

Formal effect measures could not be applied, due to included studies being case series, case reports, cross-sectional and cohort studies.

### Synthesis methods

This study did not include a control group; therefore, the findings were contextualized by examining the frequency of reported symptoms. Clinical presentation, potential impact on fertility, and imaging and biopsy findings were also reviewed.

Statistical analyses were performed using SPSS. Given the included studies were naturally heterogeneous, statistical heterogeneity (I^2^) was not calculated, and narrative synthesis was preferred to summarize the results. Funnel plot analysis was also not applicable due to study type heterogeneity.

### Reporting bias assessment

Formal bias assessment was not applicable due to study heterogeneity; however, potential bias was considered a limitation.

### Certainty assessment

Certainty of the evidence was inferred from methodological quality scores using the JBI critical appraisal checklists. Given that the included studies were predominantly case reports, case series, case–control studies and cohort studies, the certainty of evidence is inherently limited due to substantial risks of selection, publication, confounding and measurement bias. Therefore, the overall certainty of evidence across outcomes should be interpreted as low to very low.

## Results

### Study selection

The search for this review was conducted in PubMed, Web of Science, Google Scholar and Cochrane Library. After removing duplicates and irrelevant studies, 110 records remained. Following title and abstract screening, 72 studies were excluded because specific urological data were not reported. A full-text review was performed for 38 studies, which were included in this review [6–10, 12, 13, 16–46] (Fig. 1).

### Study characteristics

The included studies comprised 14 case reports, 10 case series, 10 cross-sectional studies and 4 cohort studies.

Studies were published between 1973 and 2025, and data were derived from multiple countries, predominantly Türkiye and Israel. In total, 2040 FMF patients were reported, all of whom had at least one documented urological manifestation related to FMF (Table 1).

**Table 1.** Characteristics of included studies and JBI scores

Study name	Study citation	Reference	Study type	Country	Number of patients	JBI score
Acute scrotal pain complicating familial Mediterranean fever in children	Eshel G, Vinograd I, Barr J, Zemer D. Acute scrotal pain complicating familial Mediterranean fever in children. Br J Surg 1994 Jun; 81(6):894–6. doi: 10.1002/bjs.1800810633. PMID: 8044614	[6]	Case series	Israel	29	5/9
Evaluation of bladder function in children with familial mediterranean fever and outcomes: a retrospective study	Yener S, Türkmen Ş, Ağaçli MO, Ilçe Z, Sözeri B. Evaluation of bladder function in children with familial mediterranean fever and outcomes: a retrospective study. BMC Urol 2025 Apr 12; 25(1):89. doi: 10.1186/s12894-025-01777-9. PMID: 40221701; PMCID: PMC11993960	[7]	Cohort	Türkiye	51	9.5/11
Acute orchitis in familial Mediterranean fever	Eshel G, Zemer D, Bar-Yochai A. Acute orchitis in familial Mediterranean fever. Ann Intern Med 1988 Jul 15; 109(2):164–5. doi: 10.7326/0003-4819-109-2-164. PMID: 3382109	[8]	Case series	Israel	13	6.5/9
Value of testis biopsy in the diagnosis of systemic amyloidosis	Ozdemir BH, Ozdemir OG, Ozdemir FN, Ozdemir AI. Value of testis biopsy in the diagnosis of systemic amyloidosis. Urology 2002 Feb; 59(2):201–5. doi: 10.1016/s0090-4295(01)01510-2. PMID: 11834385	[9]	Case series	Türkiye	72	8.5/9
Testicular function in patients with familial Mediterranean fever on long-term colchicine treatment	Levy M, Yaffe C. Testicular function in patients with familial Mediterranean fever on long-term colchicine treatment. Fertil Steril 1978 Jun; 29(6):667–8. doi: 10.1016/s0015-0282(16)43342-x. PMID: 658478	[10]	Case series	Israel	47	3.5/9
Reduction in sperm output by febrile attacks of familial Mediterranean fever: a case report	French DJ, Leeb CS, Jecht EW. Reduction in sperm output by febrile attacks of familial Mediterranean fever: a case report. Fertil Steril 1973 Jun; 24(6):490–3. doi: 10.1016/s0015-0282(16)39739-4. PMID: 4710010	[12]	Case report	USA	1	5/8
Safety of biologic therapy in kidney and liver transplant recipients with systemic inflammatory diseases: a real-world study from Israel	Furer V, Kersh O, Berman M, Grupper A, Rabinowich L, Peleg H, Pokroy-Shapira E, Elkayam O. Safety of biologic therapy in kidney and liver transplant recipients with systemic inflammatory diseases: a real-world study from Israel. Rheumatology (Oxford) 2025 Jun 3:keaf303 doi: 10.1093/rheumatology/keaf303. Epub ahead of print. PMID: 40459901	[13]	Cohort	Israel	76	10/11
Acute orchitis in recurrent polyserositis	Moskovitz B, Bolkier M, Nativ O. Acute orchitis in recurrent polyserositis. J Pediatr Surg 1995 Oct; 30(10):1517–8. doi: 10.1016/0022-3468(95)90428-x. PMID: 8786510	[16]	Case report	Israel	1	5/8
The first case of familial Mediterranean fever associated with renal amyloidosis in Korea	Koo KY, Park SJ, Wang JY, Shin JI, Jeong HJ, Lim BJ, Lee JS. The first case of familial Mediterranean fever associated with renal amyloidosis in Korea. Yonsei Med J 2012 Mar; 53(2):454–8. doi: 10.3349/ymj.2012.53.2.454. Erratum in: Yonsei Med J 2012 Jul 1; 53(4):873. Erratum in: Yonsei Med J 2012 May; 53(3):670. PMID: 22318840; PMCID: PMC3282977	[17]	Case report	South Korea	1	6.5/8
Acute scrotal involvement in children with familial Mediterranean fever	Gedalia A, Mordehai J, Mares AJ. Acute scrotal involvement in children with familial Mediterranean fever. Am J Dis Child 1992 Dec; 146(12):1419–20. doi: 10.1001/archpedi.1992.02160240029009. PMID: 1456246	[18]	Case report	Israel	1	5.5/8
Familial Mediterranean fever in the differential diagnosis of pediatric acute scrotum	Makay BB, Kefi A, Unsal E. Familial Mediterranean fever in the differential diagnosis of pediatric acute scrotum. Ege J Med 46.2 (2007):101–3	[19]	Case report	Türkiye	1	6/8
A rare case of bladder amyloidosis complicating familial Mediterranean fever	Suchartlikitwong S, Lapumnuaypol K, Getzug T. A rare case of bladder amyloidosis complicating familial Mediterranean fever: 979. Am J Gastroenterol 110 (2015):S422–3	[20]	Case report	Thailand	1	7.5/8
The association of familial Mediterranean fever and polyarteritis nodosa: a case report	Shiari R, Sadat Ahadi H, Farivar S, Sayyahfar S. The association of familial Mediterranean fever and polyarteritis nodosa: a case report. Arch Pediatr Infect Dis 2015; 3(2):e17469. doi : 10.5812/pedinfect.17469	[21]	Case report	Iran	1	7.5/8
Azoospermia in familial Mediterranean fever patients: the role of colchicine and amyloidosis	Ben-Chetrit E, Backenroth R, Haimov-Kochman R, Pizov G. Azoospermia in familial Mediterranean fever patients: the role of colchicine and amyloidosis. Ann Rheum Dis 1998 Apr; 57(4):259–60. doi: 10.1136/ard.57.4.259. PMID: 9709191; PMCID: PMC1752571	[22]	Case report	Israel	1	4.5/8
Recurrent episodes of acute scrotum with ischemic testicular necrosis in a patient with familial Mediterranean fever	Livneh A, Madgar I, Langevitz P, Zemer D. Recurrent episodes of acute scrotum with ischemic testicular necrosis in a patient with familial Mediterranean fever. J Urol 1994 Feb; 151(2):431–2. doi: 10.1016/s0022-5347(17)34973-x. PMID: 8283547	[23]	Case report	Israel	1	7.5/8
Secondary bladder amyloidosis with familial Mediterranean fever in a living donor kidney transplant recipient: a case report	Imamura S, Narita S, Nishikomori R, Tsuruta H, Numakura K, Maeno A, Saito M, Inoue T, Tsuchiya N, Nanjo H, Heike T, Satoh S, Habuchi T. Secondary bladder amyloidosis with familial Mediterranean fever in a living donor kidney transplant recipient: a case report. BMC Res Notes 2016 Oct 19; 9(1):473. doi: 10.1186/s13104-016-2273-2. PMID: 27760547; PMCID: PMC5070197	[24]	Case report	Japan	1	7/8
Familial Mediterranean fever as an unusual cause of acute scrotum	Lausch E, Fisch M, Beetz R. Familial Mediterranean fever as an unusual cause of acute scrotum. J Urol 2001 Apr; 165(4):1262–3. PMID: 11257698	[25]	Case report	Germany	1	8/8
A rare presentation of familial Mediterranean fever; acute scrotum and hydrocele amyloidosis	Yilmaz R, Ozer S. A rare presentation of familial mediterranean fever; acute scrotum and hydrocele amyloidosis. Iran J Pediatr 2010 Sep; 20(3):367–9. PMID: 23056732; PMCID: PMC3446044	[26]	Case report	Türkiye	1	7/8
The effect of colchicine treatment on spermatozoa: a cytogenetic approach	Kastrop P, Kimmel I, Bancsi L, Weima S, Giltay J. The effect of colchicine treatment on spermatozoa: a cytogenetic approach. J Assist Reprod Genet 1999 Oct; 16(9):504–7. doi: 10.1023/a : 1020511318806. PMID: 10530407; PMCID: PMC3455632	[27]	Cross sectional	Netherlands	2	6.5/8
Azoospermia due to testicular amyloidosis in a patient with familial Mediterranean fever	Haimov-Kochman R, Prus D, Ben-Chetrit E. Azoospermia due to testicular amyloidosis in a patient with familial Mediterranean fever. Hum Reprod 2001 Jun; 16(6):1218–20. doi: 10.1093/humrep/16.6.1218. PMID: 11387295	[28]	Case report	Israel	1	7/8
NOD2/CARD15 gene mutations in patients with familial Mediterranean fever	Berkun Y, Karban A, Padeh S, Pras E, Shinar Y, Lidar M, Livneh A, Bujanover Y. NOD2/CARD15 gene mutations in patients with familial Mediterranean fever. Semin Arthritis Rheum 2012 Aug; 42(1):84–8. doi: 10.1016/j.semarthrit.2011.12.002. Epub 2012 Jan 12. PMID: 22244368	[29]	Cross sectional	Israel	402	8/8
Fertility in male patients with familial Mediterranean fever and paternal effect of FMF on pregnancy outcomes and complications	Parlar K, Azman FN, Sıcakyüz SL, Rızaoğlu M, Azman E, Yüzbaşıoğlu MB, Korkmaz D, Uğurlu S. Fertility in male patients with familial Mediterranean fever and paternal effect of FMF on pregnancy outcomes and complications. Intern Emerg Med 2025 Apr; 20(3):797–803. doi: 10.1007/s11739-025-03881-y. Epub 2025 Feb 5. PMID: 39907917; PMCID: PMC12009244	[30]	Cross Sectional	Türkiye	180	7/8
Clinicopathological and epidemiological analysis of amyloidosis in Turkish patients	Ensari C, Ensari A, Tümer N, Ertug E. Clinicopathological and epidemiological analysis of amyloidosis in Turkish patients. Nephrol Dial Transplant 2005 Aug; 20(8):1721–5. doi: 10.1093/ndt/gfh890. Epub 2005 Jun 21. PMID: 15972323	[31]	Cross sectional	Türkiye	111	6.5/8
Coexisting diseases in patients with familial Mediterranean fever	Salehzadeh F, Enteshari Moghaddam A. Coexisting diseases in patients with familial Mediterranean fever. Open Access Rheumatol 2020 May 28; 12:65–71. doi: 10.2147/OARRR.S252071. PMID: 32547265; PMCID: PMC7266519	[32]	Cross sectional	Iran	400	5.5/8
Semen analysis in adolescents with familial Mediterranean fever	Kaya Aksoy G, Koyun M, Usta MF, Çomak E, Akman S. Semen analysis in adolescents with familial Mediterranean fever. J Pediatr Urol 2019 Aug; 15(4):342.e1–342.e7. doi: 10.1016/j.jpurol.2019.04.001. Epub 2019 Apr 5. PMID: 31036478	[33]	Cross sectional	Türkiye	72	6.5/8
Mediterranean fever gene mutation analysis in infertile Turkish males	Etem EO, Erol D, Huseyin Y, Arslan A. Mediterranean fever gene mutation analysis in infertile Turkish males. Genet Mol Res 2010 Apr 6; 9(2):611–9. doi: 10.4238/vol9-2gmr743. PMID: 20391345	[34]	Cross sectional	Türkiye	155	6.5/8
Familial Mediterranean fever-associated infertility and underlying factors	Atas N, Armagan B, Bodakci E, Satis H, Sari A, Bilge NSY, Salman RB, Yardımcı GK, Babaoglu H, Kilic L, Ozturk MA, Goker B, Haznedaroglu S, Kasifoglu T, Kalyoncu U, Tufan A. Familial Mediterranean fever-associated infertility and underlying factors. Clin Rheumatol 2020 Jan; 39(1):255–61. doi: 10.1007/s10067-019-04773-1. Epub 2019 Sep 9. PMID: 31502094	[35]	Cross sectional	Türkiye	582	8/8
The effects of long-term colchicine therapy on male fertility in patients with familial Mediterranean fever	Ehrenfeld M, Levy M, Margalioth EJ, Eliakim M. The effects of long-term colchicine therapy on male fertility in patients with familial Mediterranean fever. Andrologia 1986 Jul-Aug; 18(4):420–6. doi: 10.1111/j.1439-0272.1986.tb01801.x. PMID: 3752545	[36]	Cross sectional	Israel	19	4.5/8
The outcome of pregnancy in the wives of men with familial mediterranean fever treated with colchicine	Ben-Chetrit E, Berkun Y, Ben-Chetrit E, Ben-Chetrit A. The outcome of pregnancy in the wives of men with familial mediterranean fever treated with colchicine. Semin Arthritis Rheum 2004 Oct; 34(2):549–52. doi: 10.1016/j.semarthrit.2004.07.004. PMID: 15505771	[37]	Cohort	Israel	343	10/11
Long-term effectiveness and safety of canakinumab in pediatric familial Mediterranean fever patients	Gülez N, Makay B, Sözeri B. Long-term effectiveness and safety of canakinumab in pediatric familial Mediterranean fever patients. Mod Rheumatol 2020 Jan; 30(1):166–171. doi: 10.1080/14397595.2018.1559488. Epub 2019 Nov 21. PMID: 30556769	[38]	Cohort	Türkiye	15	10/11
Acute epididymitis in boys: evidence of a post-infectious aetiology	Somekh E, Gorenstein A, Serour F. Acute epididymitis in boys: evidence of a post-infectious aetiology. J Urol 2004 Jan; 171(1):391-4; discussion 394. doi: 10.1097/01.ju.0000102160.55494.1f. PMID: 14665940	[39]	Case series	Israel	44	9/9
The acute scrotum in Arab children with familial Mediterranean fever	Majeed HA, Ghandour K, Shahin HM. The acute scrotum in Arab children with familial Mediterranean fever. Pediatr Surg Int 2000; 16(1–2):72–4. doi: 10.1007/s003830050019. PMID: 10663841	[40]	Case series	Jordan	16	8.5/9
Familial Mediterranean fever in children: a single centre experience in Jordan	Al-Wahadneh AM, Dahabreh MM. Familial Mediterranean fever in children: a single centre experience in Jordan. East Mediterr Health J 2006 Nov; 12(6):818–23. PMID: 17333828	[41]	Case series	Jordan	56	8.5/9
Spectrum of renal involvement in familial Mediterranean fever	Said R, Hamzeh Y, Said S, Tarawneh M, al-Khateeb M. Spectrum of renal involvement in familial Mediterranean fever. Kidney Int 1992 Feb; 41(2):414–9. doi: 10.1038/ki.1992.57. PMID: 1552714	[42]	Case series	Jordan	15	6/9
Novel use of interleukin-1 antagonists in male familial Mediterranean fever patients with infertility: case series	Egeli B, Parlar K, Filiz B, Durucan I, Ugurlu S. Novel use of interleukin-1 antagonists in male familial Mediterranean fever patients with infertility: case series. Arch Rheumatol 2024 Aug 26; 39(3):474–475. doi: 10.46497/ArchRheumatol.2024.10269. PMID: 39507831; PMCID: PMC11537677	[43]	Case series	Türkiye	2	7/9
Nutcracker syndrome: a potentially underdiagnosed cause of proteinuria in children with familial Mediterranean fever	Avar-Aydin PO, Ozcakar ZB, Cakar N, Fitoz S, Karakas HD, Yalcinkaya F. Nutcracker syndrome: a potentially underdiagnosed cause of proteinuria in children with familial Mediterranean fever. Pediatr Nephrol 2022 Jul; 37(7):1615-1621. doi: 10.1007/s00467-021-05337-9. Epub 2021 Nov 18. PMID: 34796389	[44]	Case series	Türkiye	576	9/9
No appreciable decrease in fertility in Behçet’s syndrome	Uzunaslan D, Saygin C, Hatemi G, Tascilar K, Yazici H. No appreciable decrease in fertility in Behçet’s syndrome. Rheumatology (Oxford) 2014 May; 53(5):828–33. doi: 10.1093/rheumatology/ket436. Epub 2013 Dec 24. PMID: 24369417	[45]	Cross sectional	Türkiye	36	8/8
Rapid progressive glomerulonephritis in patients with familial Mediterranean fever	Said R, Hamzeh Y, Tarawneh M, el-Khateeb M, Abdeen M, Shaheen A. Rapid progressive glomerulonephritis in patients with familial Mediterranean fever. Am J Kidney Dis 1989 Nov; 14(5):412–6. doi: 10.1016/s0272-6386(89)80176-3. PMID: 2816934	[46]	Case report	Jordan	1	8/8

This table summarizes study names, authors, published year, study type, country, number of patients, JBI scores and quality. JBI: Joanna Briggs Institute.

**Table 2.** Summary of urological manifestations in FMF patients

Category	Manifestation	No. of patients	**Mean ± s.d.** **age (years)**		Male sex (%)	Key clinical features	Diagnostics	Main outcomes	References
Urological syndromes	Acute scrotum	64	8.5 ± 4.31		100	Recurrent attacks (75%)	USG (in 5 patients)	Hospitalization in 40.6%	[6, 16–19, 23, 25, 26, 29, 40]
						Tenderness (59.3%)	CT (in 1 patient)	Exploration done in 29.6%	
						Swelling (59.3%)	Nuclear scans (in 2 patients)	Testicular ischaemia/necrosis in 1.5%	
						Erythema (48.4%)	Biopsies (in 4 patients): no consistent findings		
						Epididymal enlargement (6.2%)			
						Urethral discharge 1.5%			
						Associated:			
						Epididymo-orchitis (4.6%)			
						UTIs (3.1%)			
						Testicular torsion (6.2%)			
						Hydrocele (6.2%)			
						Trauma (3.1%)			
						Testicular amyloidosis (1.5%)			
	Epididymo-orchitis	25	14.59 ± 10.69		100	Orchitis (72%)	USG (in 3 patients)	Acute scrotum in 12%	[8, 13, 16, 19, 23, 39, 42, 46]
						Epididymitis (4%)		Hydrocele in 12%	
						Epididymo-orchitis (24%)		Spermatic cord oedema in 12%	
						Fever (84%)		Ischaemia/necrosis in 4%	
						Abdominal pain (28%)		Recurrent episodes in 2 patients	
						Rash (4%)			
	Testicular torsion	4	9.6 ± 3.1		100	Acute scrotum (100%)	Laboratory: elevated WBC	Six hospitalization total in 4 patients	[6]
						Tenderness (100%)			
						Scrotal erythema (50%)			
						Swelling (25%)			
						Trauma (25%)			
	Hydrocele	4	12 ± 5.09		100	Left-sided (50%)Bilateral (25%)Unspecified unilateral (25%)Fever (25%)	USG	Surgeries performed in 50%:	[16, 19, 23, 26]
								Hydrocele repair in 25%	
								Radical orchiectomy for necrosis in 25%	
								Spontaneous remission in 25%	
						Testicular pain (25%)			
						Fever + pain (25%)			
						Acute scrotum (100%)			
						Epididymo-orchitis (75%, contralateral)			
						UTIs (50%)			
						Scrotal infection (25%)			
						Scrotal inflammation (25%)			
						Tenderness (75%)			
						Erythema (75%)			
						Epididymal enlargement (75%)			
						Swelling (100%)			
						Testicular ischaemia/necrosis (25%)			
	UTIs	4	16.75 ± 1.89		50	Testicular pain (in male patients) (100%)	Laboratory: elevated ESR and WBC	Antibiotics treatment in 100%	[13, 23, 26]
Histology-proven amyloidosis	Testicular amyloidosis	40	N/A		100		Biopsy	Patients reported to be infertile without further data	[9, 26, 28, 31]
	Bladder amyloidosis	2	N/A		N/A		Biopsy		[20, 31]
Semen/hormone outcomes	Semen parameters	189	30.16 ± 11.93		100	N/A	Spermiogram	Azospermia in 26.9%	[10, 12, 22, 27, 28, 33, 34, 36, 43]
								Oligospermia in 14.8%	
								Oligoasthenozoospermia in 0.5%	
								Sperm concentration (*n* = 3) mean: 36 ± 12.16 million/ml	
								Sperm motility (*n* = 18) mean: 49.16 ± 26%	
								Sperm rate (*n* = 12) mean: 40.25 ± 25.6 million/ml; total sperm count (*n* = 20) mean: 53.98 ± 32.91 million/ml	
								Semen volume (*n* = 11) mean: 2.75 ± 0.68 ml	
	Hormone levels	10	N/A		100	N/A		LH elevated in 60%	[10, 22, 28]
								FSH elevated in 1 patient	
								Testosterone (total and free) within normal ranges	
								Prolactin within normal ranges	
								β-HCG within normal ranges	
								AFP within normal ranges	
								LDH within normal ranges	
Clinical infertility and partner outcomes	Infertility (12 months criteria)			N/A	100	N/A	Spermiogram	Infertile according to: sperm parameters in 5 patients	[9, 10, 12, 22, 27, 28, 30, 33, 37, 43, 45]
							Clinical diagnosis	Clinical diagnosis in 15 patients	
								Colchicine-related infertility in 7 patients	
	ART outcomes	1	N/A		100	Unable to conceive for 13 years	N/A	Failed IVF twice	[27]

This table systematically summarizes the reported urological manifestations and fertility outcomes in FMF patients. Each row corresponds to a specific manifestation. AFP: alpha-fetoprotein; ART: assisted reproductive techniques; FSH: follicle stimulating hormone; HCG: human chorionic gonadotropin; IVF: *in vitro* fertilization; LDH: lactate dehydrogenase; LH: luteinizing hormone; USG: US sonography; UTI: urinary tract infection; WBC: white blood cell. N/A: Not applicable.

### Demographic and genetic characteristics

The mean age across all included studies was 23.35 ± 16 years, with 118 patients younger than 18 years (all male) and 5 patients older than 45 years. A total of 43.2% were female, reflecting both mixed-gender cohorts and cases where bladder amyloidosis or urinary tract infections (UTIs) were reported in women. *MEFV* genotyping was reported in 70.7% of cases. The most frequent mutation was M694V, observed in 42.3% of patients. Other reported mutations included V726A (4.2%), M680I (4.2%), E148Q (3%), P368S (0.5%), R761H (0.3%), A744S (0.2%), K695R (0.1%), M694I (0.1%), F479L (<0.01%) and R202Q (<0.01%). In addition, 14.6% of patients had no detectable mutations. Familial FMF history was reported in 30.4% of cases. Amyloidosis was present in 42 patients, the bladder involvement in 2 cases and there was testicular involvement in 40 cases. All amyloidosis diagnoses were biopsy confirmed [6–10, 12, 13, 16–46].

### Urological manifestations

The spectrum of urological manifestations is summarized in Table 2.

#### Epididymo-orchitis

A total of 25 patients reported experiencing either epididymo-orchitis, orchitis or epididymitis. The mean ± s.d. age was 14.59 ± 10.69 years among patients. Eighteen (72%) patients had orchitis, one (4%) patient had epididymitis [8] and six (24%) patients had epididymo-orchitis. One patient had bilateral orchitis [16], one patient had bilateral epididymo-orchitis [42] and one patient had left-sided epididymo-orchitis [23]. No additional data were given. Two patients experienced epididymo-orchitis twice, and no further details were reported. Thirteen patients accounted for 20 episodes of orchitis, while the frequency was not specified in the remaining five. The mean age was 14.66 ± 13.11 years at the first episode.

Out of 25 patients, three (12%) presented as acute scrotum. Three (12%) patients had hydrocele on the same side as epidymo-orchitis. Three (12%) patients had spermatic cord oedema [8], and one (4%) had vascular congestion, one (4%) patient had scrotal infection and two had scrotal inflammation [23]. Four (16%) patients had scrotal tenderness, scrotal swelling and epididymal enlargement, while two (8%) had scrotal erythema. Additionally, one (4%) patient had testicular ischaemia and necrosis [23].

Twenty-one (84%) patients presented with fever, seven (28%) had abdominal pain, five (20%) had arthritis, three (12%) had chest pain, two (8%) had testicular pain and one (4%) had a rash on his ankle. Eighteen (72%) patients had elevated ESR, and other blood tests had no significant results.

USG was performed on three (12%) patients during subsequent episodes. All the USGs described enlargement of testicles and hydroceles [8, 13, 16, 19, 23, 39, 42, 46].

#### Acute scrotum

Sixty-four patients had acute scrotum. The mean age was 8.5 ± 4.31 years. A study with 39 (60.9%) patients reported that acute scrotum was mostly unilateral and on the right side, though no statistical comparison was provided [6]. However, among the researchers who specified the affected side, three (4.6%) cases involved the left side and one (1.5%) was bilateral, while none was reported on the right side. Only two patients had just one acute scrotum attack. In contrast, 48 (75%) patients had more than one attack. The mean age for the first acute scrotum attack was 5.47 ± 4.53 years.

Among 64 patients, 3 (4.6%) patients presented with epididymo-orchitis. Two (3.1%) patients had UTIs and were treated with antibiotics. Four (6.2%) patients had testicular torsion. Four (6.2%) patients had hydrocele. One (1.5%) patient had congestion on the spermatic cord vessels [23]. One (1.5%) patient had scrotal inflammation [26], and one (1.5%) patient had scrotal infection [23]. Two (3.1%) patients had scrotal trauma [6]. Thirty-eight (59.3%) patients had scrotal tenderness, 31 (48.4%) patients had scrotal erythema, 38 (59.3%) patients had scrotal swelling, 4 (6.2%) patients had epididymal enlargement and 1 (1.5%) patient had urethral discharge [6]. One (1.5%) patient had testicular ischaemia and necrosis [23]. Additionally, one (1.5%) patient had testicular amyloidosis.

Twenty-six (40.6%) patients were hospitalized due to acute scrotum, and 19 (29.6%) underwent exploratory surgery.

Five scrotal USGs, one CT scan, two nuclear imaging studies and four biopsies were performed. However, no consistent imaging findings were reported across studies [6, 16–19, 23, 25, 26, 29, 40].

#### Hydrocele

Four patients had hydrocele. The mean age for hydrocele was 12 ± 5.09 years. Hydrocele was present on the left side in two patients (50%), bilateral in one patient (25%) [26] and unilateral in one patient (25%) without specifying the side [19].

Out of four patients, one patient had fever [16], one patient had testicular pain [26] and one patient had both during FMF flares [23].

All patients presented as acute scrotum, but the side of hydrocele did not overlap, except in the bilateral case [16]. Three (75%) patients had epididymo-orchitis, and none was on the same side. Two (50%) patients had UTIs, and both were treated with antibiotics. One (25%) patient had scrotal infection [23], and one (25%) patient had scrotal inflammation [26]. Three (75%) patients had scrotal tenderness, three (75%) patients had scrotal erythema, three (75%) patients had epididymal enlargement and all four patients had scrotal swelling. Additionally, one (25%) patient had testicular ischaemia and necrosis [23].

Three (75%) patients were using colchicine, and one (25%) of them had spontaneous remission [16].

Three (75%) patients had elevated ESR, one (25%) patient had elevated CRP [26] and one (25%) patient had mild leucocytosis [16].

USG was performed in all four patients, revealing hydroceles and enlargement of the testicles. Two (50%) patients had surgeries: one (25%) of them was a hydrocele repair and the other was a radical orchiectomy for testicular necrosis [16, 19, 23, 26].

#### Urinary tract infections

Four patients presented with UTIs, two (50%) of them were female [13]. The mean age was 16.75 ± 1.89 years.

All male patients experienced testicular pain. Both patients had elevated ESR and white blood cell (WBC) levels, and were treated with antibiotics [13, 23, 26].

#### Testicular torsion

Four patients diagnosed with testicular torsion [6]. The mean age was 9.6 ± 3.1 years. WBC count was elevated in all patients. All four patients presented as acute scrotum with scrotal tenderness. One (25%) patient had scrotal trauma, one patient had scrotal swelling and two (50%) patients had scrotal erythema.

Four patients have been hospitalized a total of six times.

#### Imaging and biopsy

USG, MRI, CT and biopsy findings were reported. USG and biopsy proved effective for diagnosing patients with urological manifestations, whereas MRI and CT did not demonstrate significant diagnostic yield.

#### Sperm parameters

Semen analysis was performed in 189 patients [10, 12, 22, 27, 28, 33, 34, 36, 43]. The mean age of the patients was 30.16 ± 11.93 years. All patients had an abstinence period of at least 48 h.

Azoospermia was present in 51(26.9%) patients, oligospermia was present in 28 (14.8%) patients and oligoasthenozoospermia [27] was present in 1 (0.5%) patient. The mean age for patients who had semen analysis was 35.68 ± 5.49 years, and 10 (5.2%) of them were younger than 18 years. The youngest patients to have semen analysis were 15 years old [33]. Seventy-seven (40.7%) patients had the M694V mutation. Other reported mutations included E148Q (20.1%), V726A (13.8%), M680I (7.9%), P368S (3.7%), R761H (2.6%), A744S (2.1%), K695R (1.1%), M694I (1.1%) and F479L (0.5%). In addition, 6.3% of patients carried other or undefined variants.

Sperm concentration was reported for three patients [36], and the mean was 36 ± 12.16 million/ml. Motility was reported for 18 patients and the mean was 49.16 ± 26%; however, the studies did not specify whether this referred to total, progressive or non-progressive motility. Sperm rate was reported for 12 patients with a mean of 40.25 ± 25.6 million/ml; total sperm count was reported for 20 patients with a mean of 53.98 ± 32.91 million/ml; and semen volume was reported for 11 patients with a mean of 2.75 ± 0.68 ml.

#### Fertility

Clinically, among patients who had 12 months of unprotected intercourse without achieving pregnancy, 15 were classified as infertile. All infertile patients were using colchicine. Eleven of the infertile patients had semen analysis, and one patient, was unable to conceive for 13 years and tried assisted reproductive techniques, failed *in vitro* fertilization twice, had a sperm penetration defect and a 9.1% aneuploidy rate [36], and one patient had low motility [43]. The other nine did not show any abnormalities in their semen analysis.

Patients who had testicular amyloidosis were reported to be infertile, but no further data were given [10].

Hormone levels were evaluated for 10 patients [10, 22, 28]. Luteinizing hormone (LH) was elevated in six (60%) patients, and follicle stimulating hormone (FSH) was elevated in one [28] (10%) patient. FSH, total and free testosterone, prolactin and beta-human chorionic gonadotropin (β-HCG) hormone levels, together with alpha-fetoprotein and lactate dehydrogenase enzyme levels were within normal ranges. Only hormonal blood tests were reported. Furthermore, no patients reported any urological discomfort.

Thirty-two patients were reported for colchicine usage. Across several studies, the authors described seven (21%) patients as having ‘colchicine-related infertility’; however, these reports were based solely on the original authors’ interpretations and were not supported by analyses. Colchicine dose was reported for 22 patients, with a mean dosage of 1.5 ± 0.57 mg. Seven patients achieved 11 successful pregnancies and three miscarriages while using colchicine [9, 10, 12, 22, 27, 28, 30, 33, 37, 43, 45].

#### Surgery and hospitalization

Patients were hospitalized for urological reasons 27 times, and surgery was performed in 20 (74%) patients; 1 (3.7%) was a radical orchiectomy [23], 1 (3.7%) was a hydrocele repair [26] and 18 (66.6%) were exploratory surgeries. Mean age was 10.13 ± 3.31 years.

In four patients (14.8%) with testicular torsion cases, a total of six hospitalizations were reported [6, 23, 26, 40].

### Risk of bias and quality assessment

All studies were evaluated using the JBI Critical Appraisal Checklist, categorized by study design. According to JBI checklists, most studies met a higher proportion of reporting criteria. However, because all included studies were case reports, case series, case–control studies or cohort studies, this should not be interpreted as a low risk of bias. These study designs carry substantial risks of selection, publication, confounding and measurement bias.

## Discussion

In this systematic review comprising 38 studies and involving 2040 FMF patients, genitourinary manifestations were found to be more diverse than previously recognized. Acute scrotum and epididymo-orchitis emerged as the most frequent presentations, while less common, yet clinically relevant conditions included testicular torsion, infertility and amyloidosis involving the testes or bladder.

Male infertility also was a notable issue. Although there are several infertility cases reported as ‘colchicine-related infertility’, current evidence does not support causality. On the basis of this review, it is uncertain whether colchicine has a direct effect on infertility. Further studies involving larger patient cohorts are necessary to better explain this relationship and establish causality.

Semen analyses performed in patients who underwent further evaluation due to suspected infertility revealed azoospermia or oligospermia in >40% of samples [10, 12, 22, 27, 28, 33, 34, 36, 43]. However, these findings only represent laboratory abnormalities and should not be interpreted as clinical infertility, which was documented in a substantially smaller number of patients.

During data extraction, several older publications used a term ‘sterility’ that does not align with contemporary medicine terminology. Current definitions designate infertility as the failure to conceive after 12 months of unprotected intercourse. To maintain consistency and avoid misclassification, all cases originally labelled as ‘sterile’ but meeting the modern criteria for infertility were reclassified accordingly. Following this reclassification, no case fulfilled the definition of sterility. This standardization ensured conceptual clarity and enabled coherent interpretation of fertility outcomes across studies.

The occurrence of scrotal and testicular manifestations in FMF suggests that several overlapping mechanisms may underlie reproductive involvement. Recurrent inflammatory flares can compromise testicular and epididymal blood flow, contributing to oedema, transient impairment of spermatogenesis or fibrosis [47]. Febrile episodes further produce a temperature-dependent decline in sperm output with recovery after resolution of systemic inflammation [12]. Systemic inflammatory activity may also interfere with endocrine regulation, and amyloid deposits can additionally disrupt local tissue architecture, compounding functional impairment [48]. At the molecular level, pro-inflammatory cytokines such as IL-6, TNF-α and nitric oxide may disrupt Sertoli-cell junctions, alter blood–testis barrier integrity and promote germ-cell apoptosis [47]. In contrast, AA amyloid infiltration represents a structurally destructive and typically irreversible process that compresses seminiferous tubules and vascular structures, as documented in biopsy-proven cases of azoospermia [9, 28]. Distinguishing potentially reversible inflammatory or febrile changes from irreversible amyloid- or ischaemia-mediated injury is clinically important when counseling patients about prognosis and fertility potential.

A notable observation was the high frequency of the M694V *MEFV* mutation, likely reflecting the predominance of Turkish and Jewish patients in our dataset, consistent with the distribution in the global population [48, 49]. Single-centre studies were common, which may have further narrowed genetic variation and over-represented certain mutation profiles. Although M694V has been linked to more severe FMF phenotypes [50], no study has directly associated this mutation with specific urological outcomes. In particular, infertility cases were rarely accompanied by genotype data, leaving possible genotype–fertility associations unexplored.

Hormonal evaluation was carried out in only a small subset of infertile patients. Among those tested, elevated LH was the most common abnormality, suggesting a possible endocrine role. However, the limited numbers, variation in testing and incomplete reporting prevent firm conclusions from being drawn. Age patterns were also evident. Acute scrotum tended to appear in paediatric cases, whereas infertility and abnormal semen parameters were mainly evaluated in adults.

Although sperm motility was reported in several studies, none specified whether the measurement referred to total, progressive or non-progressive motility. This lack of standardization in reporting hampers comparability across studies and may partially account for the wide range of reported mean values.

Reports linking colchicine use to infertility were observational and do not allow conclusions about causality. Data on colchicine duration, relationship with infertility, reversibility and concurrent changes in FMF disease control were not reported in the primary studies and could not be evaluated. Current evidence does not support routine discontinuation of colchicine when attempting conception. Several studies have shown preserved fertility among men receiving long-term colchicine therapy [30, 36, 37], while uncontrolled FMF activity, particularly febrile or inflammatory flares, may transiently reduce sperm output or impair spermatogenesis [12, 47]. When considering all data, maintaining disease control appears clinically preferable to colchicine withdrawal.

UTIs in FMF may have been regarded as insignificant and thus have not been reported or investigated in the literature; nevertheless, we suggest that they may present a potential manifestation.

Diagnosis in these settings is not always straightforward. In FMF, acute scrotal attacks can closely mimic testicular torsion or other acute pelvic/abdominal emergencies, increasing the risk of misdiagnosis and delayed treatment [6]. Older reports lacked standardized imaging approaches, and others used less sensitive techniques than are available today, both of which could have led to underdiagnosis or misclassification.

The strength of the current evidence is modest. Most reports were case studies or small series, with incomplete reporting of key details such as age, genetic findings and diagnostic methods. Age distributions may not have been normally distributed, which could affect the interpretation of mean age values. For acute scrotum in particular, data were often descriptive, without agreed definitions or measurable criteria, making statistical pooling unfeasible.

While previous reports have described selected urological manifestations of FMF, our review demonstrates that these manifestations are more varied and clinically relevant than generally recognized. These findings are broadly in line with earlier reports [51]. Yet our synthesis offers more detailed insights into fertility outcomes, semen quality and the scarce but important biopsy-confirmed cases of testicular amyloidosis.

A number of biases likely influenced the available evidence. In the first place, publication bias is expected, as unusual, severe or biopsy-confirmed cases such as testicular amyloidosis or acute scrotal presentations are more likely to be reported than mild or self-limited manifestations. Second, many included studies originated from tertiary referral centers, which causes survivor and referral biases that overrepresent complicated or diagnostically challenging patients. Third, substantial heterogeneity exists across decades regarding imaging modalities, semen analysis protocols, and definitions of acute scrotum, epididymo-orchitis or infertility. These differences limit comparability between studies and reduce the certainty of the findings.

Our review has its limitations. Restricting the search to English-language articles from four major databases introduces the risk of selection bias. We did not search grey literature, meaning rare cases may have been missed. Even so, study screening was performed independently by four reviewers, with final inclusion decisions confirmed by two reviewers through consensus.

From a clinical perspective, FMF flares should be evaluated in greater detail concerning urogenital manifestations. It should be kept in mind that abdominal pain attacks may mask scrotal pain. Therefore, even if patients do not report such symptoms, scrotal pain and other possible manifestations should be carefully addressed during medical history taking. These findings suggest that urological manifestations in FMF deserve more attention and highlight the need for symptom-driven assessment in FMF. Clinicians should consider urgent scrotal ultrasonography in FMF patients presenting with acute scrotal pain, swelling or tenderness in order to differentiate promptly epididymo-orchitis from testicular torsion, and avoid treatment delays. In patients with recurrent scrotal episodes or unexplained infertility, hormonal evaluation and semen analysis should be considered, alongside genetic testing where appropriate. Cystoscopy or bladder imaging is warranted in those with lower urinary tract symptoms or suspected amyloidosis. Multidisciplinary collaboration among rheumatologists, urologists and nephrologists is essential to ensure timely diagnosis and provide patient-centered management.

Priorities for future research include:

- Large, prospective cohort studies to establish incidence and risk factors, as current evidence is mostly from small, retrospective reports that limit accurate risk estimation.
- Genotype–phenotype correlation studies, particularly involving M694V and other *MEFV* variants, since mutation-specific effects on urological manifestations remain unexplored.
- Consensus-based diagnostic criteria are needed for acute scrotum and epididymo-orchitis in FMF, to reduce variability in diagnosis and allow meaningful comparisons between studies.
- Long-term follow-up studies are required to assess progression and treatment outcomes of FMF-related urological manifestations, because the natural history and impact of therapies on these manifestations are poorly documented.
- Controlled studies evaluating the effects of colchicine and other treatments on fertility, hormonal profiles and semen parameters, are required to determine whether current regimens prevent or exacerbate reproductive issues.

Addressing these gaps will facilitate earlier recognition, more targeted management, and ultimately better outcomes for patients with FMF.

## Supplementary Material

keag127_Supplementary_Data

## Data Availability

The data underlying this article are derived from previously published studies, which are cited within the manuscript. No new primary data were generated. The extracted and synthesized data supporting the findings of this study are available from the corresponding author upon reasonable request.
