# Supplementary material for: Urological manifestations in familial Mediterranean fever excluding renal amyloidosis: a systematic review
Source: Rheumatology (Oxford). 2026 Mar 24;65(5):keag127. doi: 10.1093/rheumatology/keag127 (PMC13176456; doi:10.1093/rheumatology/keag127)
Supplement: keag127_Supplementary_Data [file keag127_supplementary_data.docx]

Supplementary Table S1

Pubmed:

("Familial Mediterranean Fever"[MeSH Terms] OR "FMF" OR "familial Mediterranean fever"[tiab] OR "Mediterranean fever"[tiab] OR "MEFV"[tiab] OR "autoinflammatory"[tiab] OR "autoinflammatory syndrome"[tiab] OR "periodic fever"[tiab])

AND

("Urologic Diseases"[MeSH Terms] OR "Testicular Diseases"[MeSH Terms] OR "Epididymitis"[MeSH Terms] OR "Orchitis"[MeSH Terms] OR "urinary tract"[tiab] OR "bladder"[tiab] OR "urologic"[tiab] OR "epididymo-orchitis"[tiab] OR "epididymitis"[tiab] OR "orchitis"[tiab] OR "acute scrotum"[tiab] OR "testicular torsion"[tiab] OR "testis torsion"[tiab] OR "testicular infection"[tiab] OR "testis infection"[tiab] OR "infertility"[MeSH Terms] OR "sterility"[tiab] OR "subfertility"[tiab])

 Web Of Science:

TS=("Familial Mediterranean Fever" OR FMF OR "Mediterranean fever" OR MEFV OR autoinflammatory OR "autoinflammatory syndrome" OR "periodic fever")

AND

TS=("urologic disease" OR "testicular disease" OR epididymitis OR orchitis OR "urinary tract" OR bladder OR urologic OR "epididymo-orchitis" OR "acute scrotum" OR "testicular torsion" OR "testis torsion" OR "testicular infection" OR "testis infection" OR infertility OR sterility OR subfertility)

Google Scholar:

allintitle: "Familial Mediterranean Fever"  infertility OR subfertility OR sterility OR epididymitis OR orchitis OR "epididymo-orchitis" OR "acute scrotum" OR "testicular torsion" OR "testis torsion" OR "testicular disease" OR "testicular infection" OR "testis infection" OR urologic OR "urologic disease" OR bladder OR "urinary tract"

Cochrane:

"Familial Mediterranean Fever" OR FMF OR "familial Mediterranean fever" OR "Mediterranean fever" OR MEFV OR autoinflammatory OR "autoinflammatory syndrome" OR "periodic fever"

"Urologic Diseases" OR "Testicular Diseases" OR "Epididymitis" OR "Orchitis" OR "urinary tract" OR bladder OR urologic OR "epididymo-orchitis" OR epididymitis OR orchitis OR "acute scrotum" OR "testicular torsion" OR "testis torsion" OR "testicular infection" OR "testis infection" OR infertility OR sterility OR subfertility

Supplementary Table S2: Reasons of Exclusions
